# Design of Hydrogen Storage Alloys/Nanoporous Metals Hybrid Electrodes for Nickel-Metal Hydride Batteries

**DOI:** 10.1038/srep27601

**Published:** 2016-06-07

**Authors:** M. M. Li, C. C. Yang, C. C. Wang, Z. Wen, Y. F. Zhu, M. Zhao, J. C. Li, W. T. Zheng, J. S. Lian, Q. Jiang

**Affiliations:** 1 Key Laboratory of Automobile Materials (Jilin University), Ministry of Education, and School of Materials Science and Engineering, Jilin University, Changchun 130022, China

## Abstract

Nickel metal hydride (Ni-MH) batteries have demonstrated key technology advantages for applications in new-energy vehicles, which play an important role in reducing greenhouse gas emissions and the world’s dependence on fossil fuels. However, the poor high-rate dischargeability of the negative electrode materials—hydrogen storage alloys (HSAs) limits applications of Ni-MH batteries in high-power fields due to large polarization. Here we design a hybrid electrode by integrating HSAs with a current collector of three-dimensional bicontinuous nanoporous Ni. The electrode shows enhanced high-rate dischargeability with the capacity retention rate reaching 44.6% at a discharge current density of 3000 mA g^−1^, which is 2.4 times that of bare HSAs (18.8%). Such a unique hybrid architecture not only enhances charge transfer between nanoporous Ni and HSAs, but also facilitates rapid diffusion of hydrogen atoms in HSAs. The developed HSAs/nanoporous metals hybrid structures exhibit great potential to be candidates as electrodes in high-performance Ni-MH batteries towards applications in new-energy vehicles.

Recently, with increasing pressures of environmental problems and energy crisis, more and more sights have turned to new-energy vehicles^1^,^2^,^3^. A series of advanced battery technologies have been emerged for applications in this field, nickel metal hydride (Ni-MH) batteries, Li-ion batteries, proton exchange membrane fuel cells etc^4^,^5^,^6^. Ni-MH batteries were first commercialized and they were facing great challenges from Li-ion batteries over the past few years. Nonetheless, Ni-MH batteries still hold an important position in the market, especially in hybrid electric vehicles, due to their superior safety even under abuse conditions, temperature adaptability and environmental friendliness^7^,^8^,^9^. Moreover, Ni-MH batteries have also been widely used in the fields of power tools and modern military devices^10^,^11^. A moderate energy density is sufficient while a high power density is critical for these applications. To meet the marketing requirements for high-power Ni-MH batteries, one has to enhance the high rate dischargeability (HRD) properties of their negative electrode materials—hydrogen storage alloys (HSAs), which determine the power density of Ni-MH batteries. It should be noted that although supercapacitors have high power density, the low energy density at present makes it only a complement to batteries in energy storage devices^12^.

In the last two decades, various approaches have been proposed to improve the electrochemical properties of HSAs, such as composition optimization^13^, surface modification^14^,^15^, preparation techniques modification^16^, processing methods optimization^17^ etc. The surface modification has been demonstrated as an efficient approach to enhance the HRD performance of HSAs. The traditional surface modification methods, such as acid, alkaline and fluorination treatments, could dissolve rare earth oxides or hydroxides on the surface of alloy particles. The active elements (for example, Al and Mn) are also etched to form a Ni-rich porosity layer, which is useful for accelerating the electrochemical reaction rate^14^,^15^. Several groups have reported the improvements in HRD of alloy electrodes by microencapsulating alloy particles with various coating layers, Ni, Cu, Co, Ni-P, and so on, via the electroless technic^18^,^19^,^20^,^21^,^22^,^23^,^24^,^25^. It was reported that the plated Cu and Ni could act as microcurrent collectors to increase the charge transfer rate on electrode surfaces and as barriers to protect alloy particles from oxidation^18^,^19^. The Ni-Co deposits could work as catalysts to improve the electric catalytic activity of HSAs^23^. Moreover, the electroless plating of Ni-P nanoparticles on alloy surfaces has been demonstrated to be beneficial for decreases of contact resistance and charge transfer resistance of the electrode^25^. Mechanical milling has also been introduced to improve the HRD of hydride electrodes^26^. Moreover, carbon nanotube, graphite, and nickel nanoparticles were added into HSAs to form hybrid electrodes^27^,^28^,^29^. Note that the hybrid electrode is prepared by compositing two or more different materials (in general, at least one constituent is at the nanometer scale) into a single electrode and it shows new properties not necessarily found in the individual parent components. The additives show remarkable positive effects on accelerating the absorption and desorption of hydrogen atoms, increasing the electrochemical reaction rate^27^. All these modification methods have been proved to be effective in improving the HRD performance of hydride electrodes, but the improvements are limited^26^,^27^. Moreover, they bring some disadvantages, for example, the deformation and amorphization of HSAs^26^. The current commercial Ni-MH battery in hybrid electric vehicles only deliver the specific power of 150–400 W/kg, which cannot satisfy the increasing marketing requirements^8^,^30^. The Department of Energy of US has set a target of over 650 W/kg^31^. Thus, it is urgent to explore the intrinsic factor that dominates the HRD properties of HSAs, and to develop novel materials to achieve the above target.

Conventionally, the metal hydride electrodes for electrochemical measurements are prepared by cold pressing the mixture of HSA powders and copper or nickel powders (as current collector and binder)^27^. In this regard, a large contact resistance (*R*_C_) between current collector and active materials, or within active materials, a large charge transfer resistance (*R*_CT_) and a large mass transport resistance (*R*_MT_) exist during the electrochemical reaction of electrodes^32^. The large internal resistances lead to large polarization and poor electrochemical properties. Recently, nanoporous metals have demonstrated potential technological advantages to reduce the above internal resistances due to their unique porous structures with large surface/volume ratio^32^,^33^, which have been successfully used in supercapacitors^34^, electrochemical catalysts^35^,^36^, Li-ion batteries^37^ and other energy storage devices^38^. The HRD performance of a metallic hydride electrode is mainly determined by two steps: the electrochemical reaction on the surface of alloy electrode (charge transfer process) and the diffusion of absorbed hydrogen into the alloy bulk (hydrogen diffusion process)^39^. These two steps are closely related to exchange current density (*I*_0_), electrochemical impedance and hydrogen diffusion coefficient (*D*_H_). A higher *I*_0_ value corresponds to a better kinetics of absorbed and desorbed hydrogen, a smaller polarization resistance and a faster charge transfer process^40^. A larger *D*_H_ allows for a faster transportation of absorbed hydrogen and a faster hydrogen diffusion process. Nanoporous metals have unique porous structures with large surface/volume ratio. Hence, they could be used to (i) increase the electrochemical active area and thus to improve electrochemical reaction kinetics; and (ii) decrease the internal resistance and polarization and thus to enhance the conductivity of a metallic hydride electrode. Integrating nanoporous metals with HSAs powders is expected to enhance the HRD performance of HSAs. This approach has not been reported before and it opens a door to applications of nanoporous metals in Ni-MH batteries.

The major aim of this work is to develop a new type of hybrid electrode (HSAs/NPNi) by incorporating HSAs powders with a current collector of three-dimensional bicontinuous nanoporous Ni for applications in Ni-MH batteries. Such a composite shows excellent HRD performance where its discharge capacity is 2.4 times that of the bare HSAs at a discharge current density of 3000 mA g^−1^. It is found that the NPNi introduced into the composite decreases *R*_C_ and *R*_CT_, and accelerates the diffusion rate of hydrogen atoms in HSAs. The findings in this work provide a new strategy to improve the HRD performance of Ni-MH batteries, which can also be utilized to design other electrode materials.

## Results and Discussions

**Figure 1:**
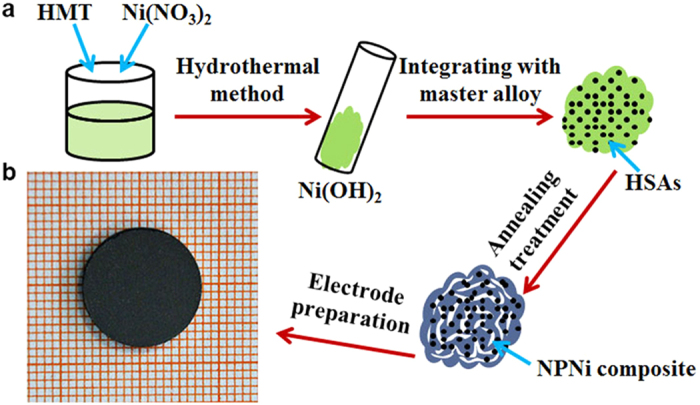
A schematic illustration of the preparation of HSAs/NPNi composite by using a top-down method. (**a**) The preparation process of the HSAs/NPNi composite. (**b**) The photograph of an electrode pellet for electrochemical measurements.

**Figure 2:**
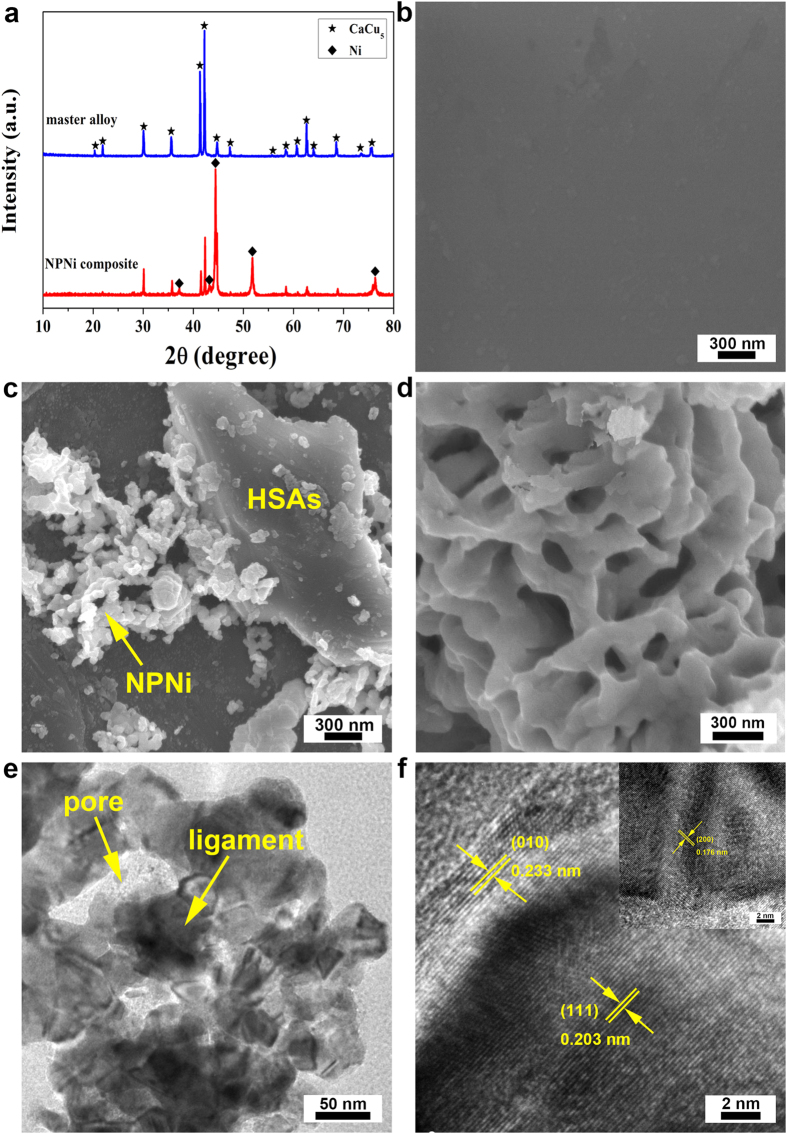
Morphological and microstructure characterizations of the master alloy and HSAs/NPNi composite. (**a**) XRD patterns of the master alloy and HSAs/NPNi composite. (**b**–**d**) are top-view SEM images of surfaces of the master alloy, composite and NPNi, respectively. (**e**,**f**) (including the inset) are TEM and HRTEM images of NPNi.

The HSAs/NPNi hybrid electrode pellets were fabricated with a facile procedure, which involves hydrothermal synthesis of Ni(OH)_2_, integration of Ni(OH)_2_ with the master alloy (LaCe)_0.9_Y_0.1_Ni_3.7_Co_0.75_(MnAl)_0.65_, thermal reduction of Ni(OH)_2_ by annealing treatment of the composites under an Ar/H_2_ atmosphere, and cold pressing the mixture of as-prepared composites and carbonyl nickel powders (Fig. 1a,b). Figure 2a shows X-ray diffraction (XRD) patterns of the master alloy and the composite. It is evident that the master alloy retains a typical CaCu_5_-type crystalline structure with a space group of P6/mmm^41^. The XRD pattern of the composite shows characteristic peaks of Ni besides the LaNi_5_ phase. Moreover, it is found that the peaks of Ni phase are broadened, which is attributed to the formation of nanoscale polycrystals [NPNi as demonstrated by scanning electron microscope (SEM) and transmission electron microscope (TEM) images below] during the annealing treatment^42^. The weight percent of NPNi in the composite is 36.87% as measured by an inductively coupled plasma (ICP) analyzer. Note that the weight percent (*y*) of original Ni (of master alloy) in the composite is determined by comparing with the contents of La, Ce, Y, Co, Mn and Al. For example, the Co content in the composite was measured by ICP as *x* (=6.48%) and we have *y* = 5.01*x* = 32.47% (the weight percent ratio of Ni to Co in the master alloy is 5.01). The total Ni content in the composite is *z* = 69.34% as measured by ICP. Then, the weight percent of NPNi in the composite (*z*-*y* = 36.87%) is obtained. The morphologies of as-fabricated samples are characterized by a field-emission SEM (FESEM) and a TEM. Figure 2b shows an SEM image of the master alloy, where clean surfaces could be observed. On the contrary, as shown in Fig. 2c, the master alloy particle is surrounded by NPNi in the composite, exhibiting rough surfaces. The NPNi with pore size of ~200 nm and ligament size of ~120 nm is formed during the reduction of Ni(OH)_2_ (Fig. 2d). Figure 2e shows a TEM image of NPNi at a low magnification. Combining Fig. 2d,e, we can find that (1) NPNi has a three-dimensional bicontinuous nanostructure; and (2) NPNi ligaments show a hyperboloid-like shape, where a positive curvature forms columnar ligaments while a negative curvature accommodates nanopores^43^,^44^. Note that the nanopores and ligaments in nanoporous metals or intermetallics are topologically and morphologically equivalent, i.e., they are inverses in a three-dimensional space, resulting in a near-zero surface curvature on average. Such a structure is typically called bicontinuous structure due to its continuous nanopore channels and ligaments. Figure 2f presents a high-resolution TEM (HRTEM) image of NPNi, in which the interplanar spacings of 0.203, 0.233 and 0.176 nm correspond to (111), (010) and (200) (inset of Fig. 2f) planes of Ni, respectively.

**Figure 3:**
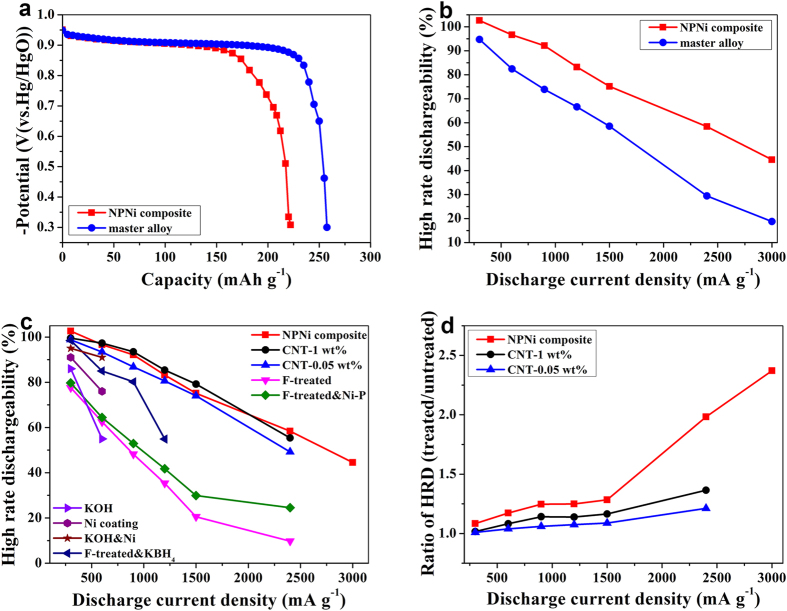
Discharge capacities and HRD properties. (**a**) The discharge capacity curves of the HSAs/NPNi hybrid electrode and master alloy electrode at room temperature. (**b**) The HRD properties of the HSAs/NPNi hybrid electrode and master alloy electrode at different discharge current densities measured at room temperature. (**c**) Comparisons of HRD properties of the HSAs/NPNi composite and reported experimental data of AB_5_-type HSAs from other advanced surface modification methods, including carbon nanotube (CNT) doping (CNT-1 wt% and CNT-0.05 wt%) for MmNi_3.6_Co_0.7_Al_0.3_Mn_0.4_^27^, fluoridation treatment (F-treated), fluoridation and the following treatment with KBH_4_ (F-treated & KBH_4_) for MlNi_3.8_Co_0.75_Mn_0.4_Al_0.2_^15^, combination of fluoridation treatment and elelctroless plating with Ni-P coating (F-treated & Ni-P) for LaNi_4_Al^25^, hot alkaline treatment at 100 °C (KOH), electroless with nickel coating (Ni coating), and combination of alkaline treatment and electroless with Ni coating (KOH & Ni) for MmX_4.3_(Al_0.3_Mn_0.4_)_0.5_ (X = Co, Mo, Mn, Al, Cu)^14^. (**d**) A comparison of ratios of HRD performance after treatment to that of untreated HSAs between NPNi incorporation and CNT doping.

Figure 3a shows the discharge capacity curves of the master alloy electrode and HSAs/NPNi hybrid electrode at a discharge current density of 60 mA g^−1^ (0.2C). The measured maximum discharge capacity *C*_max_ values are 257.51 mAh g^−1^ and 222.14 mAh g^−1^ for the master alloy and composite electrodes, respectively, which are listed in Table 1. It is known that the reactions of a metal hydride electrode during charging are typically described by (1) the Volmer reaction: H_2_O + e^−^ → H_ads_ + OH^−^; (2) the transition of hydrogen atoms between adsorbed state and absorbed state on the surface of alloy particles: H_ads_ → H_abs_; and (3) the following phase transformation process: MH_α_ → MH_β_^31^. Note that the order of the above reactions is on the contrary during the discharging process. Apart from these reactions, there is also a hydrogen evolution reaction (HER) by the Volmer-Tafel reaction route (H_2_O + e^−^ → H_ads_ + OH^−^ and the following 2H_ads_ → H_2_) or by the Volmer-Heyrovsky reaction route (H_2_O + e^−^ → H_ads_ + OH^−^ and the following H_ads_ + H_2_O  +  e^−^ → H_2_ + OH^−^) during charging^45^. It has been demonstrated that NPNi could act as a catalyst to accelerate the HER reaction^46^. As a result, during charging, H_ads_ as a product of the Volmer reaction fast recombined to generate H_2_ in the composite, which results in (1) a decrease of the number of H_ads_ to diffuse into the HSAs to create hydrides; and (2) a reduction of the utilization rate of active materials. In this case, during discharging, the reactant H_ads_ (H_ads_ + OH^−^ → H_2_O + e^−^) becomes less, leading to a smaller *C*_max_ value of the composite compared with the master alloy. Figure 3b shows the HRD property of the both electrodes. The corresponding electrochemical data are listed in Table 1. The capacity retention rates of the composite electrode are 102.6, 96.6, 92.1, 83.2, 75.2, 58.4 and 44.6% at the discharge current densities of 300, 600, 900, 1200, 1500, 2400 and 3000 mA g^−1^, respectively, showing better HRD performance than that of the master alloy electrode (the corresponding capacity retention rates are 94.7, 84.4, 73.9, 66.6, 58.5, 29.5 and 18.8%). At the discharge current density of 3000 mA g^−1^ (10C), the capacity retention rate of the composite is 2.4 times that of the master alloy. As noted above, the *C*_max_ value of the composite at 0.2C is lower than that of the master alloy. But the discharge capacity of the former at 10C (98.99 mAh g^−1^) is twice that of the latter (48.39 mAh g^−1^). Moreover, it should be noted that the discharge capacity (228.02 mAh g^−1^) of the composite at 1C (300 mA g^−1^) is a little larger than the capacity (222.14 mAh g^−1^) at 0.2C (60 mA g^−1^). This may result from stronger hydrogen evolution during charging at higher discharge current density (1C) than that at lower current density (0.2C)^19^. In the discharging process, the dissolved hydrogen is ionized in the electrolyte and an additional charge contributes to the overall value, causing larger discharge capacity of the composite electrode at 1C than that at 0.2C^19^. Note that their difference is very small (5.88 mAh g^−1^), which indicates that (1) most of hydrogen has escaped from the electrolyte in the open system of a half-cell test; and (2) contributions of the ionization of hydrogen to discharge capacities of the master alloy and composite electrodes are limited although the both electrodes show different catalytic activity for the HER reaction. For a comparison, we have also fabricated the electrode using bare HSAs without annealing under an Ar/H_2_ atmosphere and tested its electrochemical properties. It shows larger discharge capacity (302.62 mAh g^−1^) while poorer HRD performance (the capacity retention rate at 10C is 13.5%) compared with the master alloy and composite electrodes^47^,^48^. Figure 3c compares the HRD performance of HSAs/NPNi composite and reported experimental data of AB_5_-type HSAs from other advanced treatment methods, hot alkaline etching at 100 °C (KOH), electroless plating with Ni coating, and their combination for MmX_4.3_(Al_0.3_Mn_0.4_)_0.5_ (X = Co, Mo, Mn, Al, Cu)^14^, carbon nanotube (CNT) doping for MmNi_3.6_Co_0.7_Al_0.3_Mn_0.4_^27^, fluoridation treatment, fluoridation and the following treatment with KBH_4_ for MlNi_3.8_Co_0.75_Mn_0.4_Al_0.2_^15^, combination of fluoridation treatment and elelctroless plating with Ni-P coating for LaNi_4_Al^25^ etc. It can be seen that the HSAs/NPNi composite shows similar HRD performance as CNT-doped HSAs and notable superiority than other surface modification methods. Moreover, the incorporation of NPNi has better enhancement effect on the HRD properties of HSAs than CNT doping, especially when discharge current densities are larger than 1500 mA g^−1^ as shown in Fig. 3d, which plots the ratios of their HRD property after treatment to that of untreated HSAs. On one hand, this is owing to the excellent contact between NPNi and HSAs in our composite, which is beneficial for decreases of the internal resistance and polarization. On the other hand, the NPNi provides numerous active sites for the adsorption of OH^−^ and also accelerates transfer rates of the electron and ion due to its unique three-dimensional bicontinuous structure, both of which are beneficial for the enhanced electrochemical reaction kinetics.

**Figure 4:**
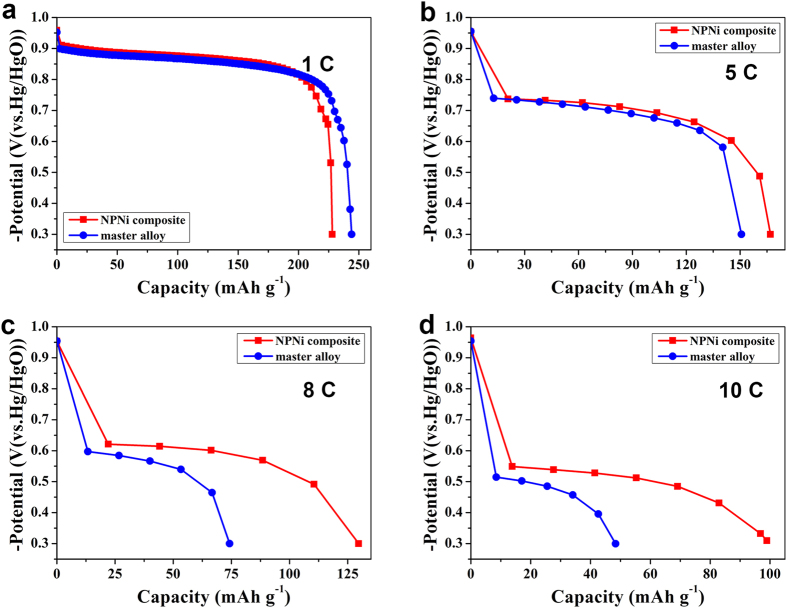
Discharge capacities of the HSAs/NPNi hybrid electrode and master alloy electrode at different discharge current densities. (**a**) 300 mA g^−1^ (1C), (**b**) 1500 mA g^−1^ (5C), (**c**) 2400 mA g^−1^ (8C) and (**d**) 3000 mA g^−1^ (10C).

Figure 4 further compares the discharge capacity curves of master alloy electrode and HSAs/NPNi hybrid electrode at different discharge current densities of 1C, 5C, 8C and 10C. It is evident that the discharge capacities of the both electrodes decrease with increasing discharge rate. Although the composite electrode has lower discharge capacity than that of the master alloy electrode at 1C, it is quite the opposite at larger discharge rates (5C, 8C and 10C), which indicates the enhanced HRD properties of the composite electrode. Moreover, the discharge potential plateau of the composite electrode is higher than that of the master alloy electrode at each discharge rate, which demonstrates higher surface activity and smaller polarization of the former^49^.

**Figure 5:**
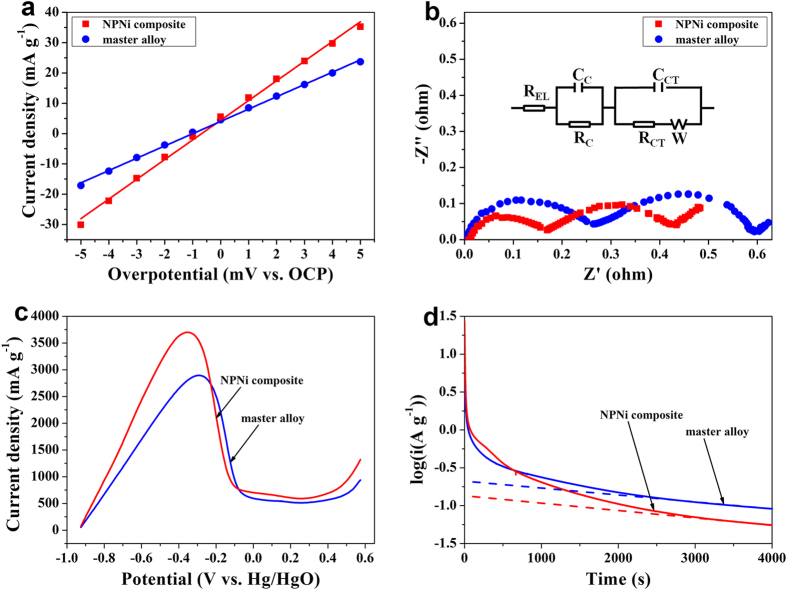
Electrochemical measurements results of the HSAs/NPNi hybrid electrode and master alloy electrode at room temperature. (**a**) Electrochemical impedance spectra at 50% DOD. (**b**) Linear polarization curves at 50% DOD. (**c**) Anodic polarization curves at 50% DOD. (**d**) Semilogarithmic curves of anodic current density versus discharge time at 100% state of charge.

To explore the nature which underpins the superior HRD performance of the HSAs/NPNi hybrid electrode compared with bare HSAs electrode, we measured their linear polarization curves, electrochemical impendence spectra (EIS), anodic polarization curves and potential steps. Figure 5a presents linear polarization curves of the both electrodes. When the overpotential *η* changes within a small range (<10 mV), i.e. at a low overpotential, *I*_0_ is given by:


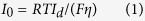


where *R*, *T*, *I*_d_ and *F* denote the gas constant, the absolute temperature, the applied current density and the Faraday constant, respectively^50^,^51^,^52^. From Table 1, the HSAs/NPNi hybrid electrode has a larger *I*_0_ value (166.88 mA g^−1^) than that of the master alloy electrode (104.22 mA g^−1^), implying better hydrogen absorption and desorption kinetics of the former. The large surface/volume ratio and the unique three-dimensional bicontinuous structure of the NPNi enlarge the contact area between active materials and the electrolyte, accelerating the mass transportation rates of OH^−^ and H_ads_^53^. The NPNi in the composites is not only a current collector, but also a bridge, which connects adjacent alloy particles to reduce the contact resistance. This can be characterized by the EIS results measured at a near-equilibrium state without damage to the samples as shown in Fig. 5b. It can be seen that each spectrum consists of two semicircles in the high-frequency region and a straight line in the low-frequency region. The small and large semicircles represent *R*_C_ and *R*_CT_, respectively, while the straight line denotes the Warburg impedance relating to the diffusion^54^,^55^. Note that the smaller the semicircle, the smaller the impedance. The *R*_C_ and *R*_CT_ values of the both electrodes can be obtained according to the equivalent circuit diagram in the figure^56^, which are listed in Table 1. It is clear that the NPNi decreases *R*_C_ and *R*_CT_, depressing the polarization (including resistance polarization and electrochemical polarization) of the hybrid electrode. This is caused by the unique three-dimensional bicontinuous nanostructure of the NPNi and the integration of NPNi and HSAs in the composite, which enable fast diffusion of reactants and products and also fast transfer of proton and electron. The above *R*_CT_ could also be used to calculate the *I*_0_ value under a small-signal alternating current^22^,^57^. The corresponding *I*_0_ function is given by:


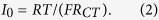


Based on Eq. (2), the calculated *I*_0_ value of the composite electrode is 387.54 mA g^−1^, which is larger than that of the master alloy electrode (*I*_0_ = 305.65 mA g^−1^). It should be noted that the calculated *I*_0_ values are much larger than those measured by the linear polarization curves. This is because the polarization resistance (*η*/*I*_d_) determined by linear polarization curves is the sum of *R*_CT_, ohmic resistance and diffusion resistance while the latter two terms cannot be neglected (they should be comparable with *R*_CT_ in this case)^58^,^59^. Nonetheless, the data obtained from the both methods indicate that the composite electrode shows larger *I*_0_ value than that of the master alloy electrode, validating the accuracy of our experimental results.

In addition to faster electrochemical kinetics, the hybrid electrode also exhibits faster hydrogen diffusion rate than that of the master alloy electrode. Figure 5c plots anodic polarization curves of the both electrodes. During the process of anodic polarization, the current density first increases with increasing overpotential and reaches a maximum value, which is defined as the limiting current density *I*_L_. The *I*_L_ value is directly proportional to the hydrogen diffusion rate in the alloy bulk, i.e., the larger the *I*_L_ value, the faster the rate of hydrogen diffusion^31^,^39^. The HSAs/NPNi hybrid electrode shows a larger *I*_L_ value (3700.98 mA g^−1^) than that of the master alloy electrode (2894.90 mA g^−1^) as listed in Table 1. Moreover, the diffusion rate of hydrogen can also be calculated by a potentiostatic method^31^ as illustrated in Fig. 5d. From the curves of discharge current density vs. discharge time, the current densities of the both electrodes decrease dramatically at first owing to the polarization (the concentration of hydrogen atoms on the alloy surface decreases obviously). Then, with increasing discharge time, the diffusion of hydrogen atoms in the alloy bulk becomes a rate-determination step and a linear relation exists between the semilogarithmic discharge current density and discharge time. In this case, the hydrogen diffusion coefficient *D*_H_ in the alloy bulk can be expressed by:


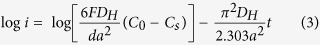


where *i*, *d*, *a*, *C*_0_, *C*_s_, *t* are the anodic current density, the alloy density, the radius of alloy particles (=25 μm here), the initial hydrogen concentration in the alloy, the surface hydrogen concentration of alloy, and the discharge time, respectively^31^,^60^. The *D*_H_ values can be determined from the slopes of lines in the figure because of the linear relations between log*i* and *t*. From Table 1, *D*_H_ of HSAs/NPNi hybrid electrode is 1.35 × 10^−10 ^cm^2 ^s^−1^, which is larger than that of the master alloy electrode (1.19 × 10^−10 ^cm^2 ^s^−1^). Thus, more H_ads_ are oxidized on the surface of the hybrid electrode, providing a larger concentration gradient to facilitate the hydrogen atoms diffusion inside the hydride^45^. Summarizing the above findings, it is found that both the faster electrochemical reaction rate on the electrode surface and faster hydrogen diffusion rate in the alloy bulk contribute to better HRD performance of the HSAs/NPNi hybrid electrode than that of the master alloy electrode.

## Conclusions

In summary, we have developed a facile and scalable strategy to prepare the HSAs/NPNi hybrid electrode, which shows superior HRD performance than that of the bare alloy electrode. The capacity retention rate of the former (44.6%) is 2.4 times that of the latter (18.8%) at a discharge current density of 3000 mA g^−1^. The unique three-dimensional bicontinuous nanostructure of NPNi in the composite substantially increases the charge transfer rate on alloy surface (higher *I*_0_ and smaller *R*_CT_) and hydrogen diffusion rate in alloy bulk (larger *I*_L_ and larger *D*_H_). Moreover, the integration of HSAs and NPNi leads to a smaller *R*_C_, effectively depressing the electrochemical polarization. The extraordinary performance of the hybrid electrode makes it a promising candidate for applications in high-power Ni-MH batteries. The top-down method in this work also provides a new strategy to enhance the HRD performance of other energy storage devices.

## Methods

Figure 1 shows a schematic diagram of the procedure to prepare the HSAs/NPNi hybrid electrode pellet. The fabricating of the composite is carried out by using a top-down method, which involves hydrothermal synthesis of Ni(OH)_2_^61^, integration of Ni(OH)_2_ with the master alloy (LaCe)_0.9_Y_0.1_Ni_3.7_Co_0.75_(MnAl)_0.65_, and the following annealing treatment, as illustrated in Fig. 1a. During the annealing process under an Ar/H_2_ atmosphere, Ni(OH)_2_ is reduced to nanoporous Ni since Ni(OH)_2_ + H_2_ → Ni + H_2_O. Moreover, the annealing treatment also strengthens the interaction between the master alloy and NPNi^62^. Such a protocol can also be generalized towards the design and production of other HSAs/nanoporous metal composites. The electrode pellet with a diameter of 15 mm (Fig. 1b) is fabricated by cold pressing the mixture of as-prepared composite and carbonyl nickel powders. Typically, the electrode pellet is tied to a Ni-coated steel strip for electrochemical measurements^39^. The experimental details are introduced below.

The master alloy (LaCe)_0.9_Y_0.1_Ni_3.7_Co_0.75_(MnAl)_0.65_ was prepared by inductive melting rare earth elements La, Ce, Y (99.5%) and other metallic elements Ni, Co, Mn, Al (99.9%) at an atmosphere of high-purity argon followed by annealing at 1000 °C for 5 h. As-annealed ingot was mechanically ground to powders. The average particle diameter of the powders is 50 ± 10 μm, which was measured by Malvern particle analyzer Mastersizer 2000. Ni(OH)_2_, the precursor of NPNi, was fabricated by a simple hydrothermal method by adding the mixture of 1.45 g Ni(NO_3_)_2_, 1.4 g hexamethylenetetramine (HMT) and 35 ml ultrapure water into a Teflon-lined stainless autoclave^61^. After that, the sealed autoclave was heated in an electric oven at 100 °C for 10 h. After the autoclave was cooled to room temperature (25 °C), green products Ni(OH)_2_ were collected by centrifugation. As-prepared HSAs powders and Ni(OH)_2_ were then mixed and ground in an agate mortar. Subsequently, the mixture was dried in an electric oven and thermally reduced in a tube furnace at a temperature of 400 °C for 5 h under an Ar/H_2_ atmosphere. Finally, we got black composite of HSAs and NPNi, where the mass fraction of NPNi was determined by ICP measurements. For a comparison study, the master alloy was also annealed under the same condition (at 400 °C for 5 h under an atmosphere of Ar/H_2_) as the composite prior to compacting with carbonyl nickel powders.

The morphology and microstructure of specimens were investigated by an FESEM (JSM-6700F, JEOL, 15 keV) and a TEM (Tecnai, F20, FEI, 200 keV). Composition analysis was performed using an ICP analyzer. XRD measurements were tested using a D/max2500pc diffractometer with Cu *K*_α_ radiation.

The electrode pellets with a diameter of 15 mm were prepared by cold pressing the mixture of fabricated composite (or master alloy) and carbonyl nickel powder in a weight ratio of 1:4 under a pressure of 8 MPa. The electrochemical measurements were performed by using an Arbin BT-2000 battery test system at room temperature in a standard tri-electrode system, which was composed of a metal hydride electrode as working electrode, a sintered Ni(OH)_2_/NiOOH electrode as counter electrode, and a Hg/HgO electrode as reference electrode, the electrolyte is 30% KOH solution. The electrodes were charged for 7.5 h at 60 mA g^−1^ (the selected over-charging ratio of 50% is to ensure full charging of the MH electrode^63^,^64^,^65^), and then discharged at 60 mA g^−1^ to a cut-off potential of −0.3 V (vs. Hg/HgO). The maximum discharge capacity *C*_max_ was obtained after 4-cycle activation. After that, the electrodes were charged at 300 mA g^−1^ for 1.5 h (the over-charging ratio is 50%) and then discharged at 300, 600, 900, 1200, 1500, 2400, 3000 mA g^−1^ to a cut-off potential of −0.3 V (vs. Hg/HgO), respectively, to measure the discharge capacities (*C*_d_) of electrodes at different discharge densities. The HRD performance is evaluated by:


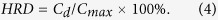


The electrochemical curves measurements were performed by using an IVIUM electrochemical analyzer at room temperature. EIS measurements were carried out in the frequency range from 100 kHz to 5 mHz with an amplitude of 5 mV vs. the open circuit potential (OCP) at 50% depth of discharge (DOD). The linear polarization curves measurements were conducted over the potential range from −5 to 5 mV (vs. OCP) at a scan rate of 0.05 mV s^−1^. The anodic polarization curves were measured by sweeping the potential from 0 to 1.5 V (vs. OCP) at a scan rate of 5 mV s^−1^ at 50% DOD. The potentiostatic experiments were performed at a potential step of +500 mV (vs. Hg/HgO) for 4000 s at 100% state of charge^13^,^64^. Note that a higher potential of +500 mV was chosen to shorten the discharging time for obtaining a linear relationship between log*i* and *t*.

## Additional Information

**How to cite this article**: Li, M. M. *et al.* Design of Hydrogen Storage Alloys/Nanoporous Metals Hybrid Electrodes for Nickel-Metal Hydride Batteries. *Sci. Rep.* **6**, 27601; doi: 10.1038/srep27601 (2016).

## Figures and Tables

**Table 1:** Electrochemical properties of the HSAs/NPNi hybrid electrode and master alloy electrode.

Samples	*C*_max_ (mAh g^−1^)	HRD_3000_ (%)	*R*_C_ (mΩ)	*R*_CT_ (mΩ)	*I*_0_ (mA g^−1^)	*I*_L_ (mA g^−1^)	*D*_H_ (×10^−10 ^cm^2 ^s^−1^)
HSAs/NPNi	222.14	44.6	157	265	166.88	3700.98	1.35
master alloy	257.51	18.8	256	336	104.22	2894.90	1.19
